# Supplementary material for: RNA sequencing-based exploration of the effects of far-red light on lncRNAs involved in the shade-avoidance response of D. officinale
Source: PeerJ. 2021 Feb 12;9:e10769. doi: 10.7717/peerj.10769 (PMC7883695; doi:10.7717/peerj.10769)
Supplement: Supplemental Information 1 [file peerj-09-10769-s001.zip › Supplemental Information/Figure S1.docx]

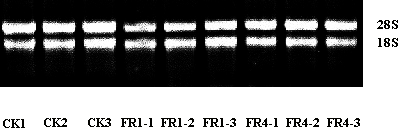


**Figure S1 The electrophoretogram of total RNA of *D. officinale* samples under different light treatments**. The clear 18S bands and 28S bands of *D. officinale* samples could be distinguished, which indicated the purity of total RNA of each sample was good.
