# Supplementary material for: RNA sequencing-based exploration of the effects of far-red light on lncRNAs involved in the shade-avoidance response of D. officinale
Source: PeerJ. 2021 Feb 12;9:e10769. doi: 10.7717/peerj.10769 (PMC7883695; doi:10.7717/peerj.10769)
Supplement: Supplemental Information 1 [file peerj-09-10769-s001.zip › Supplemental Information/Figure S3.docx]

**Figure S3 The Melt curve plots of lncRNA and targets**. (A) MSTRG.58590.1; (B) MSTRG.22972.1; (C) MSTRG.63384.1; (D) MSTRG.29691.1; (E) MSTRG.25820.7; (F) MSTRG.26377.2; (G) MSTRG.40100.1; (H) MSTRG.19522.1; (I) MSTRG.38867.1; (J) MSTRG.66273.1; (K) MSTRG.48624.1; (L) MSTRG.71071.1; (M) *TAA*1; (N) *CCD*4; (O) *ZSD*1; (P) *GGT*1_5; (Q) *CYP*90*A*1; (R) *IAA*; (S) *ARR-A*; (T) gene-MA16_Dca007905; (U) *PHYA*1; (V) *COP*1; (W) *HY*5; (X) *PIF*3.
