# Supplementary material for: RNA sequencing-based exploration of the effects of far-red light on lncRNAs involved in the shade-avoidance response of D. officinale
Source: PeerJ. 2021 Feb 12;9:e10769. doi: 10.7717/peerj.10769 (PMC7883695; doi:10.7717/peerj.10769)
Supplement: Supplemental Information 1 [file peerj-09-10769-s001.zip › Supplemental Information/Table S1.docx]

**Table S1** **The concentration and A260/A280 ratios of total RNA of samples**

| **sample** | **CK1** | **CK2** | **CK3** | **FR1-1** | **FR1-2** | **FR1-3** | **FR4-1** | **FR4-2** | **FR4-3** |
| --- | --- | --- | --- | --- | --- | --- | --- | --- | --- |
| **Concentration**  **(ng** **µl^-1^)** | 554.8 | 509.8 | 510.5 | 550.4 | 568.1 | 563.4 | 547.6 | 532.2 | 519.6 |
| **A260/A280** | 2.04 | 2.03 | 2.05 | 2.05 | 2.04 | 2.01 | 2.05 | 2.08 | 2.06 |
