# Supplementary material for: RNA sequencing-based exploration of the effects of far-red light on lncRNAs involved in the shade-avoidance response of D. officinale
Source: PeerJ. 2021 Feb 12;9:e10769. doi: 10.7717/peerj.10769 (PMC7883695; doi:10.7717/peerj.10769)
Supplement: Supplemental Information 1 [file peerj-09-10769-s001.zip › Supplemental Information/Table S15.docx]

| **Table S15 Flavonoid contents of stems in *D. officinale* under different light treatments** | | | | | | | | |  |
| --- | --- | --- | --- | --- | --- | --- | --- | --- | --- |
| Light treatments | Light intensity (µmol m^-2^ s^-1^) | Photoperiod (h) | Flavonoid contents 1  (mg g ^-1^DW) | Flavonoid contents 2  (mg g ^-1^ DW) | Flavonoid contents 3  (mg g ^-1^ DW) | Average Flavonoid  contents  (mg g ^-1^ DW) | Standard deviation | Duncan (5%) | Duncan (1%) |
| CK | 200 | 12 | 14.173 | 14.470 | 12.981 | 13.875 | 0.643 | c | C |
| FR1 | 200 | 12 | 17.270 | 19.892 | 18.760 | 18.641 | 1.073 | b | B |
| FR4 | 200 | 12 | 20.964 | 19.713 | 20.070 | 20.249 | 0.526 | a | A |
