# Supplementary material for: RNA sequencing-based exploration of the effects of far-red light on lncRNAs involved in the shade-avoidance response of D. officinale
Source: PeerJ. 2021 Feb 12;9:e10769. doi: 10.7717/peerj.10769 (PMC7883695; doi:10.7717/peerj.10769)
Supplement: Supplemental Information 1 [file peerj-09-10769-s001.zip › Supplemental Information/Table S16.docx]

| **Table S16 Alkaloid contents of leaves in *D. officinale* under different light treatments** | | | | | | | | |  |
| --- | --- | --- | --- | --- | --- | --- | --- | --- | --- |
| Light treatments | Light intensity (µmol m^-2^ s^-1^) | Photoperiod (h) | Alkaloid  contents 1  (mg g ^-1^DW) | Alkaloid contents 2  (mg g ^-1^ DW) | Alkaloid contents 3  (mg g ^-1^ DW) | Average Alkaloid  contents  (mg g ^-1^ DW) | Standard deviation | Duncan (5%) | Duncan (1%) |
| CK | 200 | 12 | 30.81 | 29.93 | 29.54 | 30.094 | 0.531 | c | C |
| FR1 | 200 | 12 | 36.08 | 37.06 | 35.69 | 36.276 | 0.575 | b | B |
| FR4 | 200 | 12 | 41.11 | 42.18 | 40.91 | 41.401 | 0.558 | a | A |
