# Supplementary material for: RNA sequencing-based exploration of the effects of far-red light on lncRNAs involved in the shade-avoidance response of D. officinale
Source: PeerJ. 2021 Feb 12;9:e10769. doi: 10.7717/peerj.10769 (PMC7883695; doi:10.7717/peerj.10769)
Supplement: Supplemental Information 1 [file peerj-09-10769-s001.zip › Supplemental Information/Table S17.docx]

| **Table S17 Alkaloid contents of stems in *D. officinale* under different light treatments** | | | | | | | | |  |
| --- | --- | --- | --- | --- | --- | --- | --- | --- | --- |
| Light treatments | Light intensity (µmol m^-2^ s^-1^) | Photoperiod (h) | Alkaloid contents 1  (mg g ^-1^DW) | Alkaloid contents 2  (mg g ^-1^ DW) | Alkaloid contents 3  (mg g ^-1^ DW) | Average Alkaloid  contents  (mg g ^-1^ DW) | Standard deviation | Duncan (5%) | Duncan (1%) |
| CK | 200 | 12 | 17.34 | 16.75 | 16.12 | 16.738 | 0.498 | b | B |
| FR1 | 200 | 12 | 21.78 | 22.71 | 22.07 | 22.188 | 0.387 | a | A |
| FR4 | 200 | 12 | 22.22 | 22.71 | 21.83 | 22.255 | 0.439 | a | A |
