# Supplementary material for: RNA sequencing-based exploration of the effects of far-red light on lncRNAs involved in the shade-avoidance response of D. officinale
Source: PeerJ. 2021 Feb 12;9:e10769. doi: 10.7717/peerj.10769 (PMC7883695; doi:10.7717/peerj.10769)
Supplement: Supplemental Information 1 [file peerj-09-10769-s001.zip › Supplemental Information/Table S18.docx]

| **Table S****18 Carotenoid contents of leaves in *D. officinale* under different light treatments** | | | | | | | | |  |
| --- | --- | --- | --- | --- | --- | --- | --- | --- | --- |
| Light treatments | Light intensity (µmol m^-2^ s^-1^) | Photoperiod (h) | Carotenoid  contents 1  ((µg g ^-1^DW) | Carotenoid contents 2  (µg g ^-1^ DW) | Carotenoid contents 3  (µg g ^-1^ DW) | Average Carotenoid  contents  (µg g ^-1^ DW) | Standard deviation | Duncan (5%) | Duncan (1%) |
| CK | 200 | 12 | 1590.00 | 1590.00 | 1545.00 | 1601.67 | 51.694 | c | C |
| FR1 | 200 | 12 | 2140.00 | 2140.00 | 2215.00 | 2200.00 | 44.159 | b | B |
| FR4 | 200 | 12 | 2495.00 | 2495.00 | 2315.00 | 2400.00 | 73.824 | a | A |
