# Supplementary material for: RNA sequencing-based exploration of the effects of far-red light on lncRNAs involved in the shade-avoidance response of D. officinale
Source: PeerJ. 2021 Feb 12;9:e10769. doi: 10.7717/peerj.10769 (PMC7883695; doi:10.7717/peerj.10769)
Supplement: Supplemental Information 1 [file peerj-09-10769-s001.zip › Supplemental Information/Table S19.docx]

| **Table S19 Carotenoid contents of stems in *D. officinale* under different light treatments** | | | | | | | | |  |
| --- | --- | --- | --- | --- | --- | --- | --- | --- | --- |
| Light treatments | Light intensity (µmol m^-2^ s^-1^) | Photoperiod (h) | Carotenoid contents 1  (µg g ^-1^DW) | Carotenoid contents 2  (µg g ^-1^ DW) | Carotenoid contents 3  (µg g ^-1^ DW) | Average Carotenoid  contents  (µg g ^-1^ DW) | Standard deviation | Duncan (5%) | Duncan (1%) |
| CK | 200 | 12 | 372.500 | 395.000 | 420.000 | 395.833 | 19.401 | c | C |
| FR1 | 200 | 12 | 458.750 | 483.750 | 463.750 | 468.750 | 10.801 | b | B |
| FR4 | 200 | 12 | 518.750 | 513.750 | 528.750 | 520.417 | 6.236 | a | A |
