# Supplementary material for: RNA sequencing-based exploration of the effects of far-red light on lncRNAs involved in the shade-avoidance response of D. officinale
Source: PeerJ. 2021 Feb 12;9:e10769. doi: 10.7717/peerj.10769 (PMC7883695; doi:10.7717/peerj.10769)
Supplement: Supplemental Information 1 [file peerj-09-10769-s001.zip › Supplemental Information/Table S2.docx]

| **Table S2 Primers information used for real-time PCR analysis of *D. officinale* genes** | | | |
| --- | --- | --- | --- |
| **Gene name** | **Primer sequences（5’→3’）** | **Size（bp）** | **TM（°C）** |
| MSTRG.38867.1-F | GCATCAAGTTGTCAGCAATCCT | 128 | 60 |
| MSTRG.38867.1-R | ATTGGTGTTTGTGGCAGGAG |  |  |
| MSTRG.25820.7-F | CGACTCCAACCGATTATTGC | 102 | 60 |
| MSTRG.25820.7-R | GTTGTCCTCCAAAGCATTGC |  |  |
| MSTRG.69319.1-F | ATATTTGATGTGCCTCAAGTATT | 115 | 60 |
| MSTRG.69319.1-R | TTTACATACCTCGCCTTTCC |  |  |
| MSTRG.66273.1-F | CCATCTAATAAACCCGACCGA | 191 | 60 |
| MSTRG.66273.1-R | GTCCTCCGATGATGCTTGTTA |  |  |
| MSTRG.48624.1-F | CCCTTCTCTGAATCGGTCATAG | 176 | 60 |
| MSTRG.48624.1-R | ACGCCTCTTCTTCGTTATTCCT |  |  |
| MSTRG.29691.1-F | TGTTGGGCACATTTAGTCTCTG | 127 | 60 |
| MSTRG.29691.1-R | TCCAAGACAAAGGAAACCTGAC |  |  |
| MSTRG.71071.1-F | AGTATGGCATTAGGCATCTTGC | 107 | 60 |
| MSTRG.71071.1-R | TGAGATTGAAACCTCCTTCCAG |  |  |
| MSTRG.24660.1-F | GGGCTTCATTGTAAGGTTGTGA | 140 | 60 |
| MSTRG.24660.1-R | CAAAGGTTGACCGTGAAAGC |  |  |
| MSTRG.19522.1-F | AAGTGTTGCTCCTTTGGCTG | 130 | 60 |
| MSTRG.19522.1-R | GCCGAAACGGAAGAAAGAGA |  |  |
| PHYA1-F | ACCTCACAGTTGCCATCTGC | 108 | 60 |
| PHYA1-R | CCCAGTCTCATCATCGTCATC |  |  |
| CYP90A1-F | GCTTTGGAATGGAGCGATTAC | 120 | 60 |
| CYP90A1-R | TGTAATGAACATCGGCAACG |  |  |
| SPA1-F | AGACAGATGGTATGCCAGCC | 161 | 60 |
| SPA1-R | GGAAGAATCCTGTGTCGGAGA |  |  |
| COP1-F | GCCGTAATGATACCCTGCTG | 131 | 60 |
| COP1-R | TGACAAGTTTGGCAATAGGTCC |  |  |
| HY5-F | GGTATCAGCACAACAGGCAAG | 122 | 60 |
| HY5-R | TCATTCTGAAGGGTGGAAAGTC |  |  |
| GGT1_5-F | CTGTTGGATTGTCACTGGTCC | 127 | 60 |
| GGT1_5-R | AAGGTTCATACGGATGGCAA |  |  |
| PIF3-F | GCCAAAGGTTGATTCAGGTTC | 148 | 60 |
| PIF3-R | CTTCAGTTCTTTCAGCAGCCA |  |  |
| gene-MA16_Dca014147-F | GATTATCAAGTCCCAACTCCCA | 195 | 60 |
| gene-MA16_Dca014147-R | GCACAGATTCTTCAGAGGCAG |  |  |
| gene-MA16_Dca007905-F | GAAGGCAGGTGTTGAGGGTA | 129 | 60 |
| gene-MA16_Dca007905-R | GTTCTCTTTGTCAGCATCCACA |  |  |
| ACTIN-F | AGCCATACTGTCCCAATCTACG | 114 | 60 |
| ACTIN -R | AGCCACGCTCGGTAAGAATC |  |  |
