# Supplementary material for: RNA sequencing-based exploration of the effects of far-red light on lncRNAs involved in the shade-avoidance response of D. officinale
Source: PeerJ. 2021 Feb 12;9:e10769. doi: 10.7717/peerj.10769 (PMC7883695; doi:10.7717/peerj.10769)
Supplement: Supplemental Information 1 [file peerj-09-10769-s001.zip › Supplemental Information/Table S20.docx]

| **Table S20 Polysaccharide contents of leaves in *D. officinale* under different light treatments** | | | | | | | | |  |
| --- | --- | --- | --- | --- | --- | --- | --- | --- | --- |
| Light treatments | Light intensity (µmol m^-2^ s^-1^) | Photoperiod (h) | Polysaccharide contents 1  (mg g ^-1^DW) | Polysaccharide contents 2  (mg g ^-1^ DW) | Polysaccharide contents 3  (mg g ^-1^ DW) | Average polysaccharide  contents  (mg g ^-1^ DW) | Standard deviation | Duncan (5%) | Duncan (1%) |
| CK | 200 | 12 | 96.571 | 92.098 | 93.589 | 94.086 | 2.277 | c | C |
| FR1 | 200 | 12 | 100.618 | 101.044 | 103.387 | 101.683 | 1.491 | b | B |
| FR4 | 200 | 12 | 111.054 | 107.433 | 108.498 | 108.995 | 1.861 | a | A |
