# Supplementary material for: RNA sequencing-based exploration of the effects of far-red light on lncRNAs involved in the shade-avoidance response of D. officinale
Source: PeerJ. 2021 Feb 12;9:e10769. doi: 10.7717/peerj.10769 (PMC7883695; doi:10.7717/peerj.10769)
Supplement: Supplemental Information 1 [file peerj-09-10769-s001.zip › Supplemental Information/Table S21.docx]

| **Table S21 Polysaccharide contents of stems in *D. officinale* under different light treatments** | | | | | | | | |  |
| --- | --- | --- | --- | --- | --- | --- | --- | --- | --- |
| Light treatments | Light intensity (µmol m^-2^ s^-1^) | Photoperiod (h) | Polysaccharide  contents 1  (mg g ^-1^DW) | Polysaccharide  contents 2  (mg g ^-1^ DW) | Polysaccharide  contents 3  (mg g ^-1^ DW) | Average polysaccharide  contents  (mg g ^-1^ DW) | Standard deviation | Duncan (5%) | Duncan (1%) |
| CK | 200 | 12 | 68.669 | 66.113 | 71.651 | 68.811 | 2.772 | c | C |
| FR1 | 200 | 12 | 81.022 | 85.921 | 82.939 | 83.294 | 2.469 | a | A |
| FR4 | 200 | 12 | 81.448 | 82.300 | 76.976 | 80.241 | 2.860 | b | B |
