# Supplementary material for: RNA sequencing-based exploration of the effects of far-red light on lncRNAs involved in the shade-avoidance response of D. officinale
Source: PeerJ. 2021 Feb 12;9:e10769. doi: 10.7717/peerj.10769 (PMC7883695; doi:10.7717/peerj.10769)
Supplement: Supplemental Information 1 [file peerj-09-10769-s001.zip › Supplemental Information/Table S22.docx]

| **Table S22 SOD content of leaves in *D. officinale* under different light treatments** | | | | | | | | |  |
| --- | --- | --- | --- | --- | --- | --- | --- | --- | --- |
| Light treatments | Light intensity (µmol m^-2^ s^-1^) | Photoperiod (h) | SOD content 1  (U g^-1^) | SOD content 2  (U g^-1^) | SOD content 3 (U g^-1^) | Average SOD content  (U g^-1^) | Standard deviation | Duncan (5%) | Duncan (1%) |
| CK | 200 | 12 | 25.14 | 27.96 | 24.18 | 25.76 | 1.96 | c | C |
| FR1 | 200 | 12 | 38.12 | 39.56 | 40.11 | 39.26 | 1.03 | b | B |
| FR4 | 200 | 12 | 45.23 | 47.13 | 47.89 | 46.75 | 1.37 | a | A |
