# Supplementary material for: RNA sequencing-based exploration of the effects of far-red light on lncRNAs involved in the shade-avoidance response of D. officinale
Source: PeerJ. 2021 Feb 12;9:e10769. doi: 10.7717/peerj.10769 (PMC7883695; doi:10.7717/peerj.10769)
Supplement: Supplemental Information 1 [file peerj-09-10769-s001.zip › Supplemental Information/Table S23.docx]

| **Table S23 Relative membrane permeability of leaves in *D. officinale* under different light treatments** | | | | | | | | |  |
| --- | --- | --- | --- | --- | --- | --- | --- | --- | --- |
| Light treatments | Light intensity (µmol m^-2^ s^-1^) | Photoperiod (h) | Relative membrane permeability 1  (%) | Relative membrane permeability 2  (%) | Relative membrane permeability 3 (%) | Average Relative membrane permeability (%) | Standard deviation | Duncan (5%) | Duncan (1%) |
| CK | 200 | 12 | 33.15 | 36.84 | 35.37 | 35.12 | 1.86 | c | C |
| FR1 | 200 | 12 | 40.35 | 41.22 | 44.53 | 42.03 | 2.21 | b | B |
| FR4 | 200 | 12 | 45.89 | 46.17 | 46.62 | 46.22 | 0.37 | a | A |
