# Supplementary material for: RNA sequencing-based exploration of the effects of far-red light on lncRNAs involved in the shade-avoidance response of D. officinale
Source: PeerJ. 2021 Feb 12;9:e10769. doi: 10.7717/peerj.10769 (PMC7883695; doi:10.7717/peerj.10769)
Supplement: Supplemental Information 1 [file peerj-09-10769-s001.zip › Supplemental Information/Table S24.docx]

| **Table S24 CaM content of leaves in *D. officinale* under different light treatments** | | | | | | | | |  |
| --- | --- | --- | --- | --- | --- | --- | --- | --- | --- |
| Light treatments | Light intensity (µmol m^-2^ s^-1^) | Photoperiod (h) | CaM content 1  (ng ml^-1^) | CaM content 2  (ng ml^-1^) | CaM content 3 (ng ml^-1^) | Average CaM content  (ng ml^-1^) | Standard deviation | Duncan (5%) | Duncan (1%) |
| CK | 200 | 12 | 13.56 | 12.93 | 14.15 | 13.55 | 0.61 | b | B |
| FR1 | 200 | 12 | 14.66 | 15.18 | 16.71 | 15.52 | 1.07 | a | A |
| FR4 | 200 | 12 | 15.42 | 16.37 | 14.72 | 15.50 | 0.83 | a | A |
