# Supplementary material for: RNA sequencing-based exploration of the effects of far-red light on lncRNAs involved in the shade-avoidance response of D. officinale
Source: PeerJ. 2021 Feb 12;9:e10769. doi: 10.7717/peerj.10769 (PMC7883695; doi:10.7717/peerj.10769)
Supplement: Supplemental Information 1 [file peerj-09-10769-s001.zip › Supplemental Information/Table S3.docx]

| **Item** | **New genes** | **Differentially expressed genes** | **lncRNAs** | **Differentially expressed lncRNAs** |
| --- | --- | --- | --- | --- |
| **Number** | 3086 | 2125 | 3370 | 136 |

**Table S3 Sequencing result data statistics under different light treatments**
